# Value and Efficacy of Foley Catheter Removal of Blunt Pediatric Esophageal Foreign Bodies

**DOI:** 10.1155/2014/679378

**Published:** 2014-01-30

**Authors:** Yasin Abdurehim, Yalkun Yasin, Qu Yaming, Zhang Hua

**Affiliations:** Department of Otolaryngology, First Affiliated Hospital of Xinjiang Medical University, Urumqi 830054, China

## Abstract

*Objective*. To discuss the safety and efficacy of Foley catheter removal of blunt pediatric esophageal foreign bodies. *Methods*. Analyzing our first 17 cases of pediatric esophageal foreign bodies removed by Foley catheter method in respect of the efficacy, removal methods, and complications. We also reviewed related literature and discussed the background, current status, and technical matters that need attention of this method. *Results*. In three-year period between May 2010 and May 2013, in 16 out of 17 children blunt radiopaque foreign bodies impacted in the esophagus were successfully removed by a Foley catheter. There were no complications. In one patient, the foreign body was advanced into stomach and came out with stool 2 days later. *Conclusions*. The technique is safe, rapid, and cost-effective procedure and applicable for blunt, flat foreign bodies impacted in the esophagus.

## 1. Introduction

Children have a natural tendency to put any small objects into their mouth; occasionally these objects can be ingested in the airodigestive tract; 80% of them will be stuck in the esophagus and 20% of them will lodge in the airway. In China, no statistical data on the incidence of esophageal foreign body is available, while the American Association of Poison Control documented 182,105 incidents of foreign body ingestion by patients younger than 20 years in 1999 [1, 2]. In most cases, an impacted esophageal foreign body is an urgent medical situation. The majority of patients with esophageal foreign bodies are children. Unlike adults, the most common pediatric esophageal foreign bodies are blunt and round objects like coins, buttons, or button batteries. The conventional method involves removal of the foreign body under direct vision using a rigid esophagoscope under general anesthesia. In fact, the majority of blunt pediatric esophageal foreign bodies can be removed by a nonoperative Foley catheter removal.

In this report we describe our experience of first 17 cases removing blunt radiopaque esophageal foreign bodies by a Foley's catheter without fluoroscopic guidance.

## 2. Materials and Methods

We evaluated the safety and efficacy of the Foley catheter technique by identifying detailed records of our first 17 cases that had undergone a Foley catheter removal of blunt radiopaque foreign bodies from May 2010 to May 2013.

### 2.1. Procedure

The materials needed for foreign body removal include a number of 14 to 18 Foley catheters, a 10 mL syringe, tongue depressor, and saline water. Additionally, pediatric direct laryngoscope, rigid esophagoscope and bronchoscope, laryngeal and bronchial forceps, suction apparatus, and oxygen supply were kept ready. All procedures were performed in the examination room of the ENT inpatient department. No patient is sedated. Radiographic confirmation is obtained to find out the presence, location, and shape of the foreign bodies. Any struggling child is immobilized by wrapping securely in a towel. The balloon of the catheter is tested before insertion to make sure that it inflates symmetrically. While the catheter is inserted transorally, patients sit or are held upright position on the edge of the examination table. Children are instructed to open their mouth naturally; in uncooperative children, a tongue depressor is put intraorally and then its two sides are bristled to prevent the child from biting the catheter. The operator holds the catheter in his left hand and inserts it by his/her right hand, while advancing it inferiorly, he or she makes the action of swallowing and asks the child to follow. When the catheter passed about 20–25 cm, the balloon is inflated with 5 mL air or saline. Before the catheter is withdrawn, patients are placed in a prone oblique position with head slightly out of the edge of the examination table. The balloon expands the esophageal lumen and frees the impacted foreign body; with moderate, steady traction, it pulls the foreign body out from the esophagus; with aid of gravity, it will usually fall out of the mouth. If balloon extraction fails to dislodge the foreign body because of under inflation or because the balloon is not down beyond the foreign body, correction is made with an additional 1–3 mL air or saline in the balloon or advancing the catheter deeper. If these corrections are unsuccessful and the foreign body will not move after 3 attempts, the technique is abandoned and the patient is referred to the rigid esophagoscopic examination under general anesthesia immediately. After successful extraction, children are monitored for 30 mins and subsequently discharged. The patents were instructed to feed the child with a soft diet and to return immediately if the child has symptoms of chest pain, fever, dysphagia, bloody saliva, respiratory difficulty, or abdominal pain.

## 3. Results

A total of 17 children (9 boys and 8 girls) with blunt esophageal foreign bodies, aged 20 months to 10 years, were included in the study. The mean age was 4 years. The duration of foreign body impaction was 30 mins to 24 hours. Chest roentgenograms were obtained in all the cases to identify the shape, size, and location of the foreign body; in all the cases, the impacted foreign body was a blunt and flat radiopaque object; 16 of them were metallic objects (11 coins, 2 buttons, 1 button battery, 1 key ring, and 1 heart shaped pendant) and one of them was a round and flat stone. Foreign body was lodged in thoracic esophagus in 7 cases, at the thoracic inlet in 6 cases and in the cervical esophagus in 4 cases. We were successful in 16 cases; in 12 of them the foreign body was removed in the first attempt and in 4 of them the foreign body was extracted in the second attempt by slightly increasing the volume of the balloon. We failed in one case; this child has a heart shaped pendant in the level of 2nd thoracic vertebra; after 3 unsuccessful attempts, we abandoned this method and had a rigid esophagascopic examination subsequently under general anesthesia. In the examination, nothing abnormal has been identified in the whole esophageal lumen. An immediate fluoroscopy was given in the operation room under a C arm and the foreign body was detected in the stomach. There were no complications like bleeding or dyspnea. Except the child who has rigid esophagoscopy under general anesthesia, all patients were discharged well on the same day after the procedure.

## 4. Discussion

Rigid esophagoscopy has long been hailed as the gold standard for the removal of esophageal foreign bodies in children. If all types of esophageal foreign bodies were considered, rigid esophagoscope is the first choice of treatment; however, the subset of blunt and flat esophageal foreign bodies can be removed by a nonoperative method with a Foley catheter. Foley catheter removal of blunt pediatric esophageal foreign bodies was first reported by Bigler in 1966 [3]. However, similar method existed long ago, according to the descriptions of Paulus Aegineta, in his book “Six of Epitome of Medicine;” during the peak of the Byzantine period, foreign bodies were extracted from the esophagus by having the patient swallow a small, dry sponge on a string, allowing it to expand in the stomach and then withdrawing the sponge [4]. Bigler hypothesized in his original article that distension of the balloon of a Foley catheter filled with contrast media inferior to the impacted foreign body would dilate the esophageal lumen, free the impaction, and allow safe extraction of the blunt and flat foreign bodies under fluoroscopic monitoring. Soon after, this simple technique has been widely used. Because of no need for anesthesia, removal of blunt esophageal foreign bodies by this method has become a relatively common problem shared by radiologists, pediatric surgeons, otolaryngologists, emergency department physicians, and gastroenterologists [1]. In real practice, certain modifications were made by some scholars, like inserting the catheter transorally, replacing contrast media with saline water or air, and performing the procedure without fluoroscopy [5–8]. By these modifications, the whole procedure becomes simpler, the radioactive contaminations can be avoided and the patients and their parents' anxieties also can be eliminated. We used this modified technique to remove blunt esophageal foreign bodies and succeeded in 16 out of 17 cases.

The main critical concern about Foley catheter removal of esophageal foreign bodies was safety, because it carries certain blindness, resulting in esophageal perforation and airway compromise. However, the incidence of all complications of Foley catheter removal of blunt foreign bodies has been consistently low in all published series. Several large studies have reported the safety and efficacy of Foley catheter extraction. The reported complication rate is as low as 0–2% [9–12]. The largest survey of pediatric radiologists by Campbell and Condon included 2500 procedures with only one serious but reversible hypoxic episode [10]. Campbell and Condon described no complications in their 100 sequential patients. J. Wang and P. Wang reported no complications in 138 sequential patients [2]. The only one case of death in the literature till now, caused by aspiration of a coin during Foley catheter removal, was reported in a survey by Hawkins [9]. At the same time he reported five patients who died while undergoing esophagoscopic removal of a coin under general anesthesia. After 10 years of editing many articles on the pros and cons of Foley catheter removal, Berdon found that great differences in opinion existed among practitioners. He concluded that “those who use the technique will continue to use it and feel comfortable: those that are not sold on the technique will remain unsold” [8]. A review of 415 cases by Schunk et al. found minor complications like epistaxis and vomiting in 8 (2%) patients and 4 (1%) patients who had the major complications of mediastinitis, transient airway compromise, respiratory distress with uneventful recovery, and esophageal laceration requiring surgery [11]. Two of these complications occurred in patients with nonradiopaque foreign bodies, the third complication was caused by an impaction of unknown duration, and the fourth occurred after multiple attempts including simultaneous use of two Foley catheters. Actually, all the 4 major complications above can totally be prevented by careful patient selection and standard performance. Foley catheter method was contraindicated to the former three cases; the fourth case was entirely the result of improper handling. All of these show that, if performed under strict inclusion criteria and standard procedure, Foley catheter removal can be the first choice of treatment for blunt and flat esophageal foreign bodies. Advantages of Foley catheter method including that they are (1) easy to perform and learn; (2) safe: complication rate is as low as 0–2% and can omit the potential complications of rigid esophagoscopy; (3) efficient: success rat is 85–100%; (4) rapid: it rarely takes more than 20 minutes; (5) having no need for anesthesia: it can prevent anesthesia related complications; (6) haveing the possibility to be performed on outpatient basis. Because it can obviate the need for anesthesia and hospitalization, this method is highly cost effective. In current health expense system in our region, the mean cost for Foley catheter extraction without fluoroscopy on an outpatient basis is less than 200RMB (cost for foreign body removal is 150RMB+cost for material is 35RMB); rigid esophagoscopy under general anesthesia costs at least 4000RMB, which translates into savings of 3800RMB.

To ensure safety and to improve success rate, it is important to follow certain guidelines and observe several precautions during the procedure.

Firstly, this method should be administered only for blunt and flat radioopaque foreign bodies. Some scholars suggest that blunt non radiopaque foreign bodies also should be included in the indications of this method [7]; however, it is not easy to estimate the shape and size of this type of foreign bodies precisely, so they are preferably removed under rigid esophagoscopy. The duration of impaction should not be longer than 72 hours, because success decreased to 50% if the duration was longer than 72 hours [11]. Button batteries are an exception; because the potential voltage burn and esophageal erosion can occur as early as 4 hours after ingestion, they should be removed endoscopically as early as possible [12]. However, if the impaction is of less than 2-hour duration, Foley catheter extraction is an acceptable alternative [13, 14]. In this series we have a 4-year-old boy who had swallowed a button battery 30 mins earlier; the battery was removed by a Foley catheter in one attempt uneventfully. This method is contraindicated in the following situations: (1) if the foreign body has been impacted for more than 72 hours, (2) if the esophagus was totally obstructed by impacted foreign body, (3) if we found suspected esophageal perforation, (4) if we found multiple foreign body impaction, (5) if patient has sign of airway distress, (6) if we found sharp edged foreign bodies, (7) and if we found button batteries that impacted for more than 2 hours. Some authors noted that children younger than 1.5 years seem to be at the highest risk for esophageal edema and failure of Foley catheter extraction [15]. This may be due to the smaller caliber of the esophagus, the softer trachea that can be compressed easily, as well as the delayed diagnosis in nonverbal, and uncooperative infants. Our youngest patient was a 20-month-old child who had swallowed a flat and round stone for 4 hours; the stone was removed in one attempt with a Foley catheter. So, one should carefully consider this method to children younger than 1.5 year.

Secondly, during the procedure, it is strongly recommended to maneuver the child into a prone oblique position while withdrawing the catheter to eliminate the freed foreign body to advance toward stomach or to the airway by gravity. Aggressive traction or forceful jerking of the catheter is contraindicated. Overdistention should be avoided. At first, inflate the balloon with 4-5 mL saline, if the balloon slips past the impacted object, try again with an additional 1–3 mL saline in the balloon [14]. Stop inflation if the patient feels pain. If the impacted foreign body cannot be removed after 3 attempts, this method should be abandoned and the child should be referred to rigid esophagoscopy under general anesthesia. After 3 unsuccessful attempts in one of our cases, without obtaining another chest X-ray film, we have esophagascopic examination under general anesthesia and did not find anything abnormal in the whole esophageal lumen; subsequently we have a fluoroscopy in the operating room under a C arm and found that the impacted object was already in the stomach. The object came out with stool 2 days later. So, before esophagascopic examination, it is very important to have another chest roentgenograms to see if the object moved or passed to the stomach. In up to 16% patients, the foreign body will advance into the stomach; this is not seen as a failure given that safe passage throughout the rest of the alimentary tract can usually be assumed [1].

Thirdly, when performing this technique, it is preferable to keep pediatric laryngoscope, rigid esophagoscope and bronchoscope, suction apparatus, and oxygen supply readily available, so that we may utilize them in critical moments. We have never required any of this equipment in all of our cases; however, we think if conditions permit, this equipment will never be the fifth wheel of the coaching.
